# Weak UVB Irradiation Promotes Macrophage M2 Polarization and Stabilizes Atherosclerosis

**DOI:** 10.1007/s12265-021-10189-7

**Published:** 2021-11-22

**Authors:** Xin-Yun Li, Tao Qin, Peng-Fei Zhang, Wen-jiang Yan, Ling-Li Lei, Jiang-Ying Kuang, Hao-Dong Li, Wen-Cheng Zhang, Xiao-Ting Lu, Yuan-Yuan Sun

**Affiliations:** 1 The Key Laboratory of Cardiovascular Remodeling and Function Research, Chinese Ministry of Education, Chinese National Health Commission and Chinese Academy of Medical Sciences, The State and Shandong Province Joint Key Laboratory of Translational Cardiovascular Medicine, Department of Cardiology, Qilu Hospital, Cheeloo College of Medicine, Shandong University, Jinan, China; 2 Department of Emergency Surgery, Qilu Hospital, Cheeloo College of Medicine, Shandong University, Jinan, China; 3 Grade 2018, School of Basic Medical Sciences, Clinical Medicine (5+3), Cheeloo College of Medicine, Shandong University, Jinan, China; 4 Department of Cardiology, The Second Hospital, Cheeloo College of Medicine, Shandong University, Jinan, China

**Keywords:** Atherosclerosis, M1 macrophage, M2 macrophage, UVB, Inflammatory, Akt

## Abstract

Atherosclerosis (AS) is a chronic cardiovascular disease endangering human health and is one of the most common causes of myocardial infarction and stroke. Macrophage polarization plays a vital role in regulating plaque stability. As an important component of sunlight, ultraviolet B (UVB) has been proven to promote vitamin D and nitric oxide synthesis. This research used an AS model in ApoE^−/−^ mice to study the effects of UVB on macrophage polarization and atherosclerotic plaque stability. In vitro, UVB irradiation increased arginase-I (Arg-I, M2 macrophage) and macrophage mannose receptor (CD206) expression, while the expression of inducible nitric oxide synthase (iNOS) (M1 macrophage) and CD86 was decreased. UVB promoted Akt phosphorylation in vitro. In vivo, UVB irradiation promoted the stabilization of atherosclerotic lesion plaques, while the phenotype of M2 macrophages increased. Our research provides new evidence for UVB in preventing and treating atherosclerosis.

## Introduction

Cardiovascular disease has the highest morbidity and mortality of all diseases in the world. Atherosclerosis (AS) is a long-lasting cardiovascular disease endangering human health, and it has become one of the most common causes of myocardial infarction and stroke. Abnormal lipid metabolism and inflammation are two key factors in the formation and progression of atherosclerotic plaques. AS is generally considered an inflammatory disease [1], as inflammation plays a vital role in all stages of the atherosclerotic process [2]. Studies have shown that immune cells such as macrophages are the main inflammatory cells, and they play a key role in causing chronic inflammation of the aortic wall and participate in the occurrence and development of AS [3]. Macrophage inflammation is a common basis of physiological and pathological changes during the initiation and development of AS [4].

It is generally believed that there are two types of macrophages: M1 macrophages and M2 macrophages. Interferon-gamma (IFN-ɣ) or lipopolysaccharide can activate M1 macrophages and interleukin (IL)-13 or IL-4 can activate M2 macrophages [5–7]. They play different roles in inflammation: M1 macrophages produce proinflammatory cytokines, such as IL-6, IL-1β, and tumor necrosis factor-α (TNF-α) and M2 macrophages produce anti-inflammatory cytokines, such as IL-10 [7–9]. The proinflammatory macrophage marker inducible nitric oxide synthase (iNOS) is expressed on M1 macrophages, and the anti-inflammatory macrophage marker arginase 1 (Arg1) is expressed on M2 macrophages [10]. Therefore, considering the proinflammatory effect of M1 macrophages and the anti-inflammatory effect of M2 macrophages, promoting the polarization of M2 macrophages may provide a new way to relieve inflammation and increase AS plaque stability.

As an important component of sunlight, ultraviolet B (UVB) has a wavelength range of 280–320 nm. Although UV exposure can cause several skin diseases, UVB light has been found to promote the production of vitamin D [11], melanin [12, 13], and nitric oxide [14]. In addition, UVB radiation has also been used to treat vitiligo [15], psoriasis [16], atopic dermatitis [17], eczema [18], and cutaneous T cell lymphoma [19]. Interestingly, UVB not only causes peripheral effects but is also related to various neurobehaviors. Recent studies have shown that learning and memory can be affected by UVB exposure [20]. In recent years, UVB exposure has been found to inhibit the formation of angiotensin II-induced abdominal aortic aneurysms in mice [21]. In former studies, researchers found that 10 and 50 mJ/cm^2^ UVB irradiation activated p42/44, p38, JNK, tyrosine kinase, and PI3K signaling pathways in macrophages in vitro [22]. It has also been suggested that ERK and Akt signaling pathways play important roles in macrophage M2 polarization [23, 24]. Considering that the polarization of macrophages plays an important role in AS and inflammation, we hypothesize that UVB may induce macrophages towards the anti-inflammatory M2 phenotype to protect against AS.

To test this hypothesis, we studied the anti-atherosclerotic effect of UVB treatment on atherosclerotic apolipoprotein E-deficient (ApoE^−/−^) mice in vivo. We also studied the possible pathways of peritoneal macrophages and human monocytic Tohoku Hospital Pediatrics-1 (THP1) cells in vitro under UVB irradiation.

## Materials and Methods

### Animals

Twenty-four 8-week-old male ApoE^−/−^ mice were obtained from Charles River Laboratory (Beijing, China), and all mice were subjected to a 12-h light/12-h dark cycle. A high-fat diet (15% cocoa butter and 0.25% cholesterol) was fed to all mice in the following 16 weeks. Subsequently, the mouse backs were shaved and randomly divided into 2 groups (*n* = 12 per group) for treatment: the UVB group received UVB irradiation (G15T8E UVB lamp emits a continuous spectrum from 275 to 320 nm) of 25 mJ/cm^2^ (28 µW/cm^2^, 15 min) every other day (three times per week) for 4 weeks, and the control (CTR) group did not receive any treatment. The irradiation dose was determined on the basis of previous studies [25]. The irradiation was conducted at approximately 8:00 a.m. Mice were sacrificed after 4-week irradiation, and aortas and peritoneal macrophages were harvested. All animal protocols were approved by the Institutional Animal Care and Use Committee of Cheeloo College of Medicine, Shandong University (animal ethics number is KYLL-2021 (KS) -746).

### Peritoneal Macrophage Extraction

Pre-cooled phosphate-buffered solution (PBS) (10 ml) was injected into the abdominal cavity of the mice, while the abdomen was kneaded softly for 2 min. The PBS was drawn out and collected, and then centrifuged for 10 min at 1000 rpm. The cells were cultivated in 24-well plates at 37 °C for 2 h until they had adhered, then the cultivation holes were washed with pre-cooled PBS. The adherent cells were peritoneal macrophages [26].

### Cell Culture

THP1 cells resuspended in 1640 medium were placed into six-well plates, and phorbol 12-myristate 13-acetate (PMA) was added to the cells at 50 ng/ml [27] for 24 h, after which the UVB group cells received UVB irradiation of 25 mJ/cm^2^. The cells in the control group received no irradiation. Twenty-four hours after UVB irradiation, cells were obtained for flow cytometry and western blot analysis. For the Auto- ja Kuljetusalan Työntekijäliitto (Akt)-inhibited group, MK-2206 was added to the cells at 10 µM. The stimulus lasted 24 h.

### ELISA Analysis

IL-6, IL-1β, and TNF-α levels in serum, culture supernatant of peritoneal macrophage, and THP1-induced M0 macrophages were determined via ELISA kits (EK106/2; EK101B; EK182; EK201B/3; EK206/3; EK282/3, Multisciences (Lianke) Biotech, Co., Ltd and R&D Systems) according to the manufacturer’s protocol. After the procedure, plates were read on a spectrometer at 450 nm and 570 nm wavelength. The results were converted to numeric values by using standard curves.

### Flow Cytometry

For fluorescence-activated cell sorting analysis, 10^5^ peritoneal macrophages or THP-1-induced M0 macrophages (CTR group and UVB group) were isolated and resuspended in 100 µl PBS, and antibodies were added to the corresponding cells. Flow cytometric analysis was performed by FACSCanto II (BD Biosciences) using Flow Jo software. The following antibodies were used: PE/Cyanine7 anti-human CD206 (BioLegend, 321,124), FITC anti-human CD86 (BioLegend, 374,204), APC anti-human CD163 (BioLegend, 333,609), PE/Cyanine7 anti-mouse CD86 (BioLegend, 105,014), FITC anti-mouse CD206 (BioLegend, 141,704), PE/Cyanine7 anti-mouse CD204 (Invitrogen, 2,263,083), and PE anti-mouse CD163 (BioLegend, 156,703).

### Histopathological Staining

The aortas and hearts were taken immediately and fixed in 4% formaldehyde overnight. HE staining was used to calculate plaque area in aortic root. The dissected aortas and aortic roots were incubated with 0.5% oil red O staining solution at room temperature for 30 min, and then washed twice with purified water. Percentage of oil red O positive area in total aortic area and percentage of aortic root area were quantitatively analyzed by using Image-Pro Plus 6.0 (IPP 6.0, Media Cybernetics, Rockville, MD, USA). For histological staining, the aortic roots were embedded into the optimal cutting temperature (OCT) to make cryosections and cut into serial 6-μm cross-sections. Rabbit monoclonal antibodies against smooth muscle actin (ab5694; Abcam, USA) and rat anti-monocyte/macrophage antibodies [MOMA-2] (ab3345; Abcam, USA) were used for immunohistochemical (IHC). Horseradish peroxidase (HRP)-conjugated goat anti-rabbit, goat anti-rat, and goat anti-mouse secondary antibodies (ZSJB-BIO, China), were used in the sections incubated with primary antibodies. Picrosirius red staining was used to detect the lipid and collagen contents of the lesions. For each slide, at least three high-power field images were captured and evaluated in a blinded fashion. The slides were quantitatively analyzed by using IPP 6.0. The plaque vulnerability index was calculated according to the formula: vulnerability index = (lipid deposit% + macrophages%)/(collagen fibers% + SMCs%)[28].

### Oil Red O Staining

Primary peritoneal macrophages were plated on cover slides in 24-well plates. Two hours later, cells were washed with PBS and new medium was added into cells. Then cells were divided into two groups: the CTR group and the UVB group. In UVB group, cells received 25 mJ/cm^2^ UVB irradiation. Then 75 µg/ml OX-LDL was added into two group cells. Twenty-four hours later, cells were washed with cold phosphate-buffered saline three times and fixed with 4% paraformaldehyde for 10 min. Subsequently, 0.5% oil red O was added to cells for 10 min. Of note, oil red O needs to be filtered to remove impurities. Foam cells were observed under a microscope at × 400 magnification.

### Western Blotting

RIPA lysis buffer supplemented with a complete protease inhibitor cocktail was used to lyse cells on ice. Equal amounts of protein (10 µg) were separated with a 10% SDS-PAGE gel, and the protein was transferred to PVDF membranes (0.22 µm, Millipore). Membranes were obstructed in protein fast blocking fluid (EpiZyme, Shanghai, China) and incubated with primary antibodies against iNOS (ab129372; Abcam, Cambridge, USA), Arg-I (ab91279; Abcam), p-AKT^ser473^ (4060, CST), p-AKT^ser308^ (13038 T, CST), AKT (4685, CST), MCP-1 (81559, CST), IL-1β (12703, CST), IL-6 (12153, CST), and TNF-α (11948, CST). GAPDH (5174, CST) was used as CTR.

### RT-PCR

Real-time PCR system detects mRNA expression. Total RNA was extracted from peritoneal macrophages using TRIzol reagent (Life Technologies). For reverse transcription (RT), a PrimeScript RT reagent kit (Takara, Shiga, Japan) was used. Quantitative PCR was performed as described previously using SYBR Premix Ex Taq (Takara) and a Roche Light Cycler 480 II instrument in a 96-well plate according to the manufacturer’s protocol. The following primers were used to amplify MCP-1, IL-6, IL-1β, TNF-α, and actin.

- TNF-α-F: GCCACCACGCTCTTCTGTCT,
- TNF-α-R: TGAGGGTCTGGGCCATAGAAC;
- IL-6-F: ACAACCACGGCCTTCCCTAC,
- IL-6-R: CATTTCCACGATTTCCCAGA;
- IL-1β-F: ACCTTCCAGGATGAGGACATGA,
- IL-1β-R: AACGTCACACACCAGCAGGTTA;
- MCP1-F: TAAAAACCTGGATCGGAACCAAA,
- MCP1-R: GCATTAGCTTCAGATTTACGGGT;
- Actin-F: CCACACCCGCCACCAGTTCG,
- Actin-R: TACAGCCCGGGGAGCATCGT.

### Statistical Analysis

All data represent at least three different experiments. GraphPad Prism 5.0 (La Jolla, CA, USA) was used for statistical analysis. The values were presented as the mean ± SEM. Student’s *t*-test was used for two-group comparisons. *p* < 0.05 was considered significant.

## Results

### UVB Irradiation Decreased Aortic Plaque Area and Enhanced Atherosclerotic Plaque Stability

**Fig. 1:**
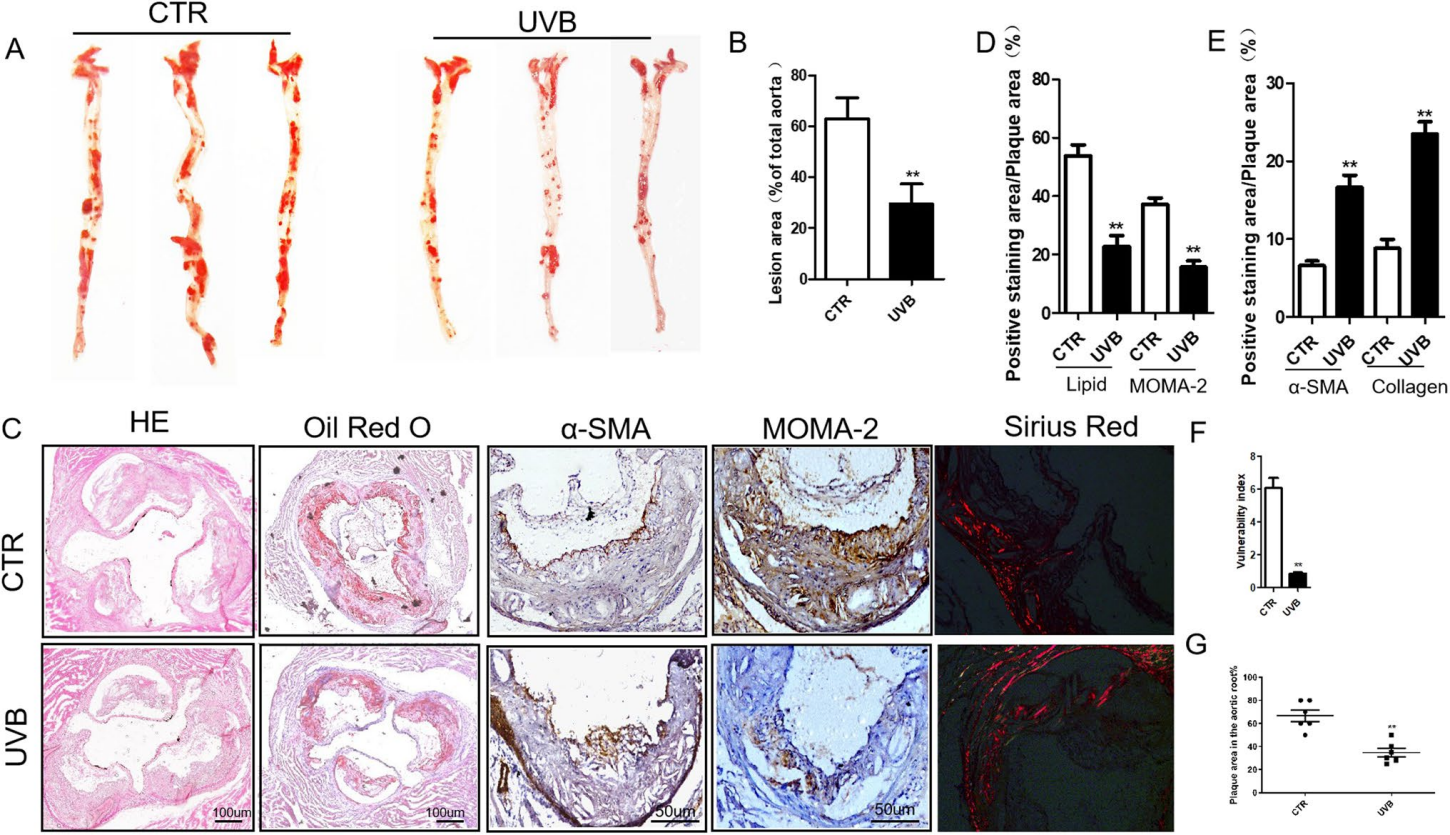
UVB obviously decreased the aortic plaque area and increased AS plaque stability in atherosclerotic ApoE-/-^− / −^ mice. A Oil red O staining of lipids in the aorta. B Statistics of A. *n* = 6. C Representative H&E, oil red O, α-SM actin, MOMA-2, and picrosirius red staining in 2 groups of ApoE ^− / − ^mice. CTR: *n* = 6. UVB: *n* = 6. D–E Relative contents of macrophages, lipids, smooth muscle cells (SMCs), and collagen in the aortic root plaques of the control and UVB groups. F Measurements of the plaque vulnerability index in the plaques of the CTR and the UVB group. G Measurements of the plaque areas in aortic roots. **p < 0.01 vs the CTR group

ApoE^−/−^ mice were fed a high-fat diet for 16 weeks to study the effect of UVB irradiation on AS in vivo. Then, the mice were divided into two groups: the CTR group and the UVB group. Mice in the UVB group received 25 mJ/cm^2^ UVB irradiation every other day (three times per week) for 1 month. The results showed that UVB irradiation decreased the aortic plaque area (Fig. 1A and B), aortic root plaque area (Fig. 1C–G) and increased the stability of aortic root plaques (Fig. 1C, D, E, and F). The characteristics of atherosclerotic vulnerable plaques are macrophage infiltration and lipid accumulation, as well as a thin cap with less collagen and smooth muscle cells (SMCs) [29, 30]. We found that after UVB treatment, the plaque content of lipids and macrophages was significantly reduced, while the plaque content of collagen and SMCs was significantly increased. These results demonstrated the beneficial effect of UVB irradiation on atherosclerosis.

### UVB Irradiation Decreased Body Weight and Inflammation But Did Not Alter the Lipid Level

**Fig. 2:**
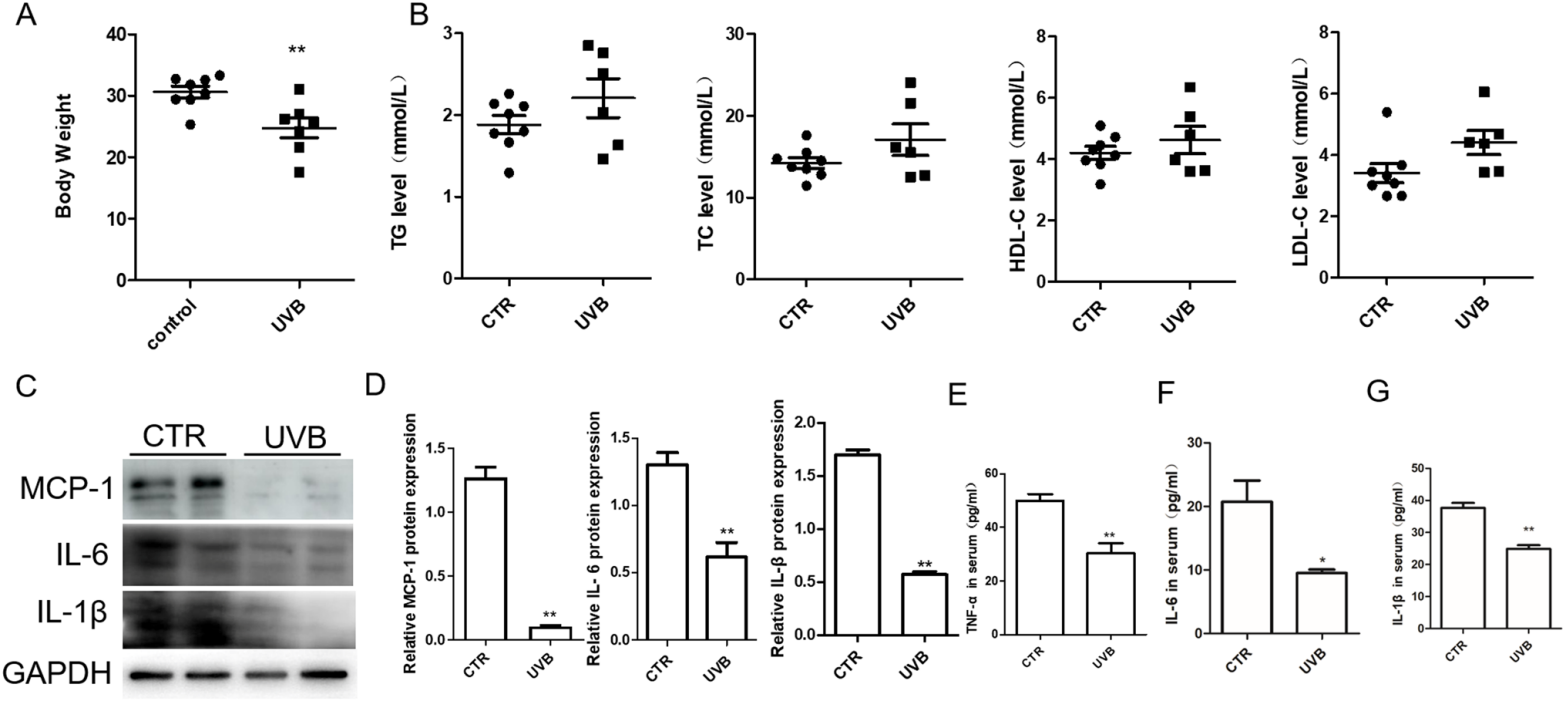
UVB irradiation decreased body weight and inflammation. A Representative body weight in two groups. B The TG, TC, HDL-C, and LDL-C level in mouse serum. C Representative protein expression of MCP-1, IL-6, and IL-1β in the aorta. *n* = 3. D Statistical analysis of C. E–G There was decreased TNF-α, IL-6, and IL-1β in UVB irradiation mouse serum. *n* = 4. **p < 0.01 vs CTR group

We found that there was decreased body weight in the UVB group (Fig. 2A). We also detected the total cholesterol (TC), total triglycerides (TG), high-density lipoprotein cholesterol (HDL-C), and low-density lipoprotein cholesterol (LDL-C) levels in mouse serum. The results showed that there were no differences between the UVB group and the CTR group (Fig. 2B). In the in vivo experiment, aortic inflammatory cytokine proteins, such as MCP-1, IL-6, and IL-1β, showed lower expression in the UVB group (Fig. 2C and D). There was decreased serum level of TNF-α, IL-1β, and IL-6 in UVB irradiation group (Fig. 2E–G).

### UVB Induced M2 Macrophage Polarization and Decreased Peritoneal Macrophage Inflammatory Cytokine Expression In Vivo

**Fig. 3:**
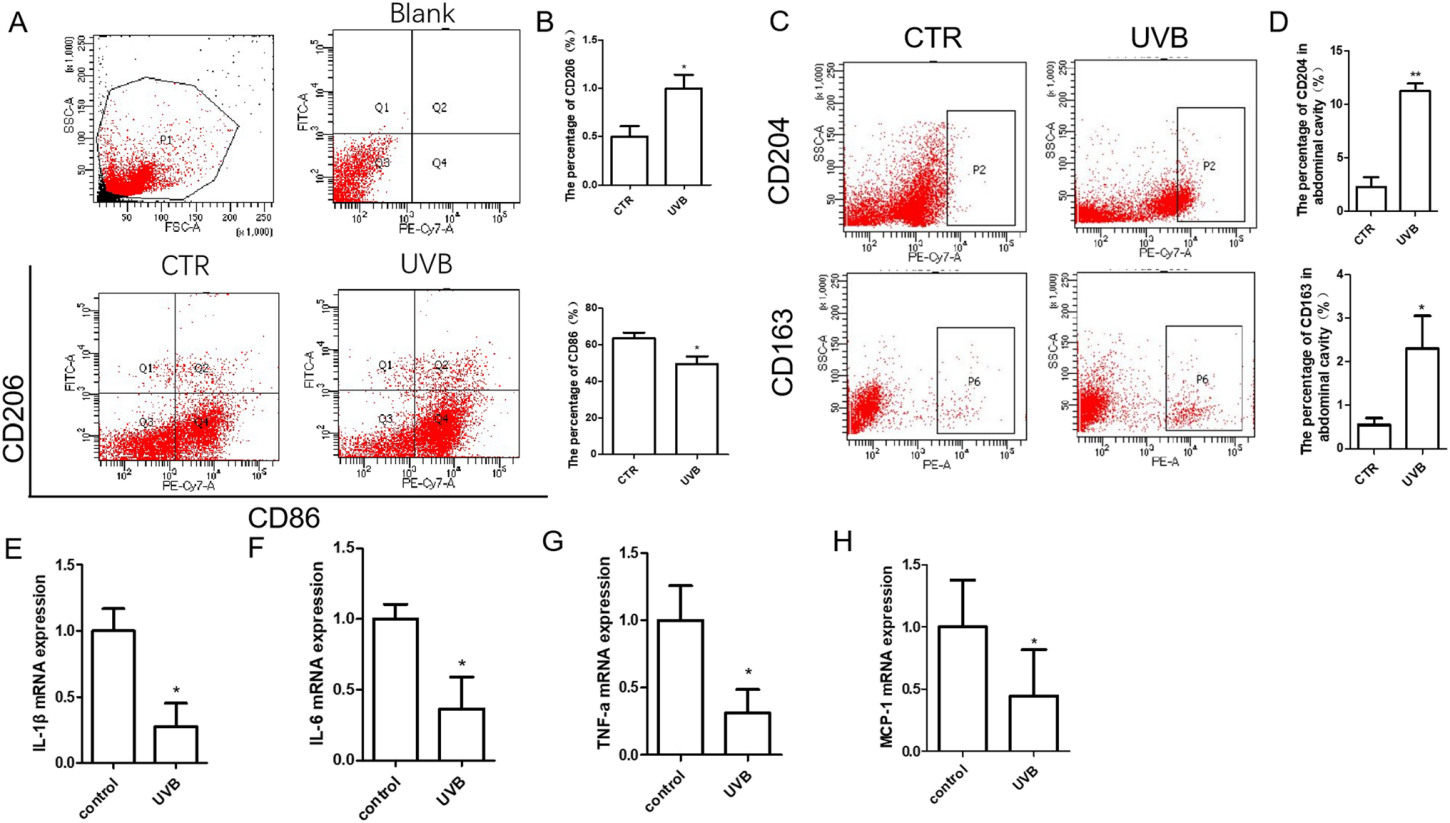
UVB irradiation increased M2 polarization of peritoneal macrophages in atherosclerotic mice. A Representative flow cytometry analysis of peritoneal macrophages in mice. B Statistical analysis of A. C Representative proportion of CD204 and CD163 positive cells in mouse peritoneal macrophages. *n* = 4 or 5. D Statistical analysis of C. E–H IL-1β, IL-6, TNF-α, and MCP-1 mRNA expression in peritoneal macrophages from the two groups. *n* = 4. *p < 0.05 vs CTR group; **p < 0.01 vs CTR

To explore whether the mouse macrophage polarization switch is involved in the preventive effect of UVB on AS, we tested the peritoneal macrophages in the CTR group and the UVB group. The results showed that the proportion of M2 phenotype (CD206 + , CD163 + , CD204 +) cells in the UVB group was higher than that in the CTR group, and the proportion of M1 phenotype (CD86 +) cells in the UVB group was much lower than that in the CTR group, as determined by flow cytometry (Figs. 3A and B, 3C–D).

Correspondingly, UVB inhibited the inflammatory response of macrophages, which manifested as a significant decrease in the mRNA of proinflammatory cytokines such as IL-1β, IL-6, MCP-1, and TNF-α in peritoneal macrophages (Fig. 3E–H).

### UVB Induced Macrophage M2 Polarization In Vitro

**Fig. 4:**
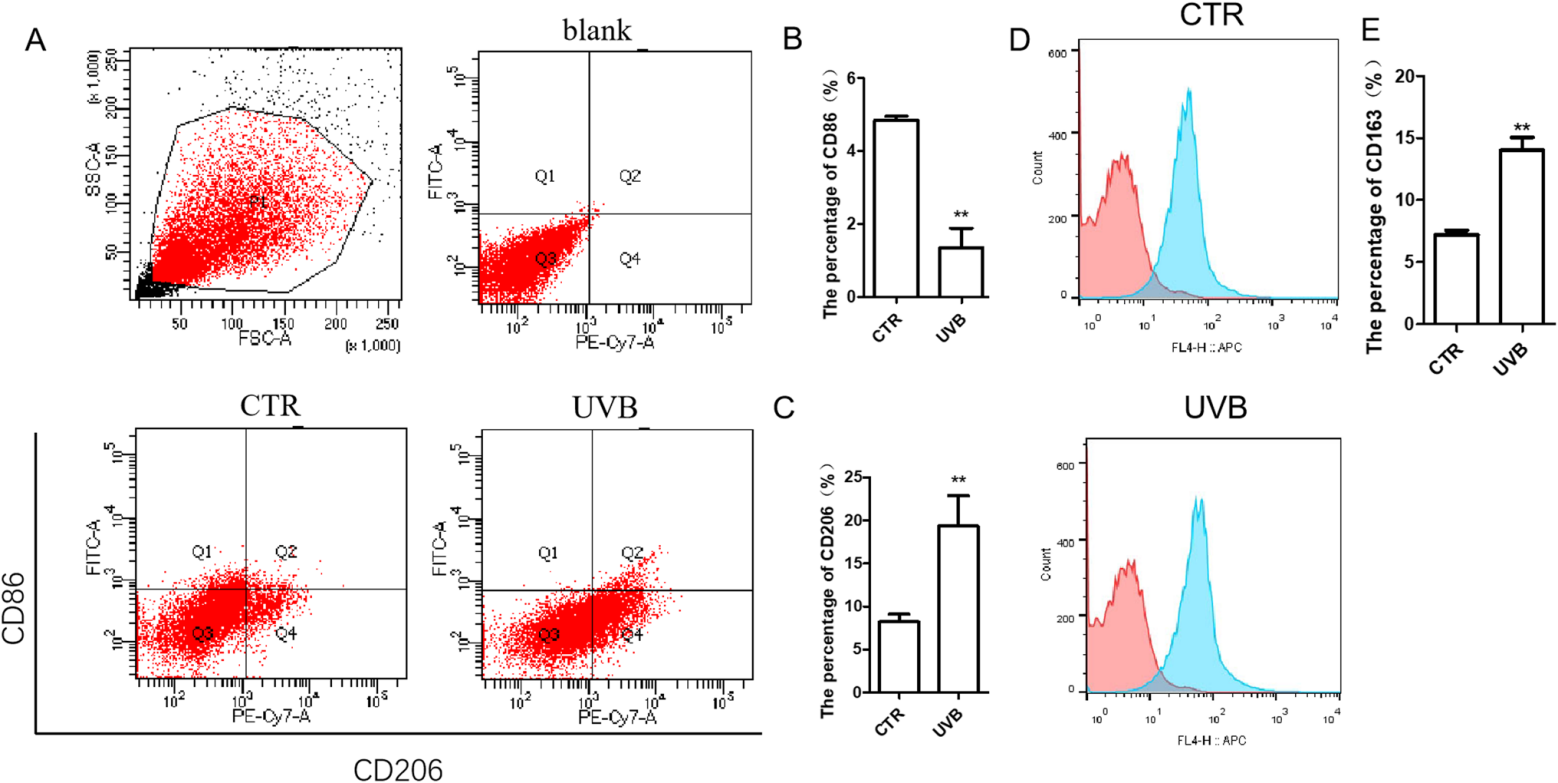
UVB irradiation induced macrophage M2 polarization in vitro. A Representative flow cytometry analysis of M0 macrophages in the CTR group and UVB group. *n* = 6. B–C Statistical analysis of A. D–E Representative proportion of CD163 positive cells in THP1 cells and statistical analysis. *n* = 5. **p < 0.01 vs CTR group

To explore the role of UVB in regulating the phenotype of macrophages in vitro, we induced THP1 cells into M0 macrophages with PMA. According to flow cytometry analysis, the percentage of M2 phenotype (CD206 + , CD163 +) cells in the UVB group was much higher than that in the CTR group, while the percentage of M1 phenotype (CD86 +) cells was much lower than that in the CTR group (Fig. 4A–E).

**Fig. 5:**
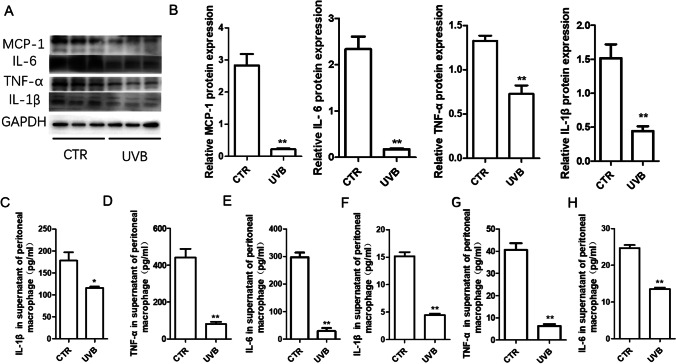
UVB irradiation decreased macrophage inflammatory cytokine expression. A Representative inflammatory cytokine protein expression of MCP-1, IL-6, TNF-α, and IL-1β. *n* = 3. B Statistical analysis of A. C–H UVB irradiation decreased IL-1β, IL-6, and TNF-α level in culture supernatant of peritoneal macrophages induced by OX-LDL or not induced by OX-LDL. *n* = 6–8. *p < 0.05 vs CTR group.**p < 0.01 vs CTR group

**Fig. 6:**
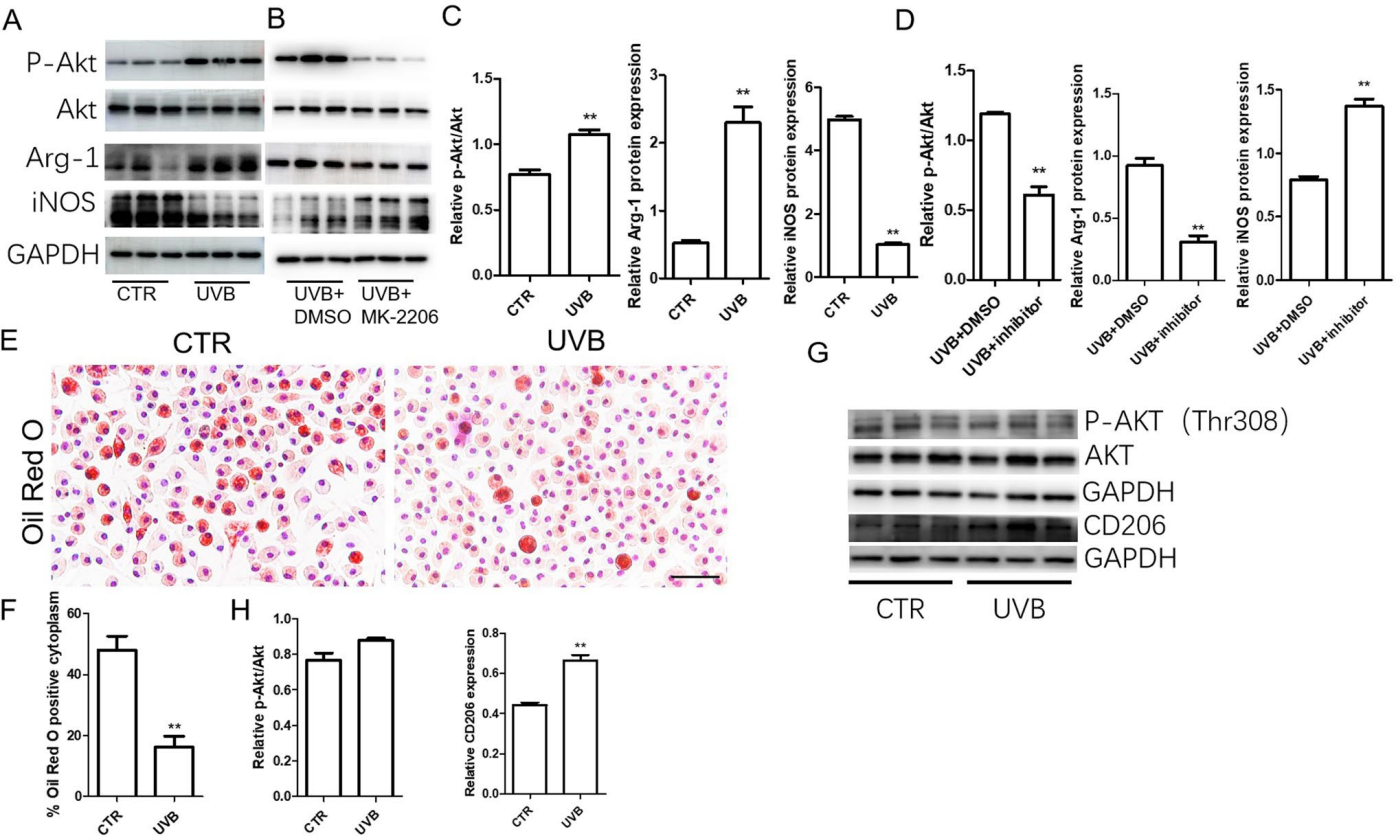
UVB induced macrophage M2 polarization through the Akt-dependent pathway. A Representative protein expression of p-Akt, Akt, Arg-1, and iNOS in the CTR group and UVB group. *n* = 3. B Representative protein expression of p-Akt, Akt, Arg-1, and iNOS in the UVB+DMSO group and UVB + MK-2206 group. *n* = 3. C Statistical analysis of A. D Statistical analysis of B. E–F Representative oil red O staining was performed to assess lipid accumulation in macrophages and statistical analysis. *n* = 5. G–H Representative protein expression of p-AKT Thr308 and CD206 in CTR and UVB-irradiated THP1 cells and statistical analysis. *n* = 3. **p < 0.01 vs CTR group. Scale bar = 50 µm

Correspondingly, UVB irradiation reduced the protein levels of MCP-1, IL-6, TNF-α, and IL-1β in THP1 cells (Fig. 5A–B). The expression of iNOS (M1 marker) and Arg-1 (M2 marker) was detected in UVB-treated or untreated M0 macrophages. We found that UVB inhibited the expression of iNOS, while the Arg-I and CD206 levels increased (Figs. 6A–C and 6G–H). UVB irradiation also decreased IL-1β, IL-6, and TNF-α expression in cell culture supernatant induced or not induced by OX-LDL in peritoneal macrophage cultured in vitro (Fig. 5C–H). Oil red O detection showed that UVB irradiation reduced macrophage foam cells induced by oxidized low-density lipoprotein (Fig. 6E–F). Therefore, these results showed that UVB can induce macrophages to acquire the anti-inflammatory M2 phenotype in vitro.

### UVB Induced Macrophage M2 Polarization Through the Akt-Dependent Pathway

Previous studies have shown that the polarization of M2 macrophages is achieved by activating Akt [24, 31–33]. To study whether Akt is involved in UVB-induced polarization of M2 macrophages, we tested the phosphorylation level of Akt in M0 macrophages induced by PMA. The results showed that the level of p-Akt/Akt in the UVB group was significantly higher than that in the CTR group. In addition, the Akt1/Akt2/Akt3 inhibitor mk-2206 significantly reversed the UVB-induced polarization of M2 macrophages and inhibited the phosphorylation of Akt ser473 (Fig. 6A–D). There was no alteration of phosphorylation of Akt Thr308 (Fig. 6G–H). These results showed that UVB-induced polarization of M2 macrophages may through an Akt-dependent pathway.

## Discussion

In this study, we confirmed that UVB promoted macrophage M2 polarization, inhibited the inflammatory response, and further promoted the stability of atherosclerotic plaques in ApoE^−/−^ mice. The effects of UVB on the polarization of M2 macrophages are mainly achieved through Akt phosphorylation.

In recent years, people have discovered that sunlight exposure has many health benefits, including protecting us from cardiovascular diseases [34], autoimmunity [35], and obesity and diabetes [36]. As one of the components of sunlight, there are an increasing number of studies on the function of UVB. Ferguson et al. found that UVB exposure can regulate the response of pro-atherosclerotic T cells by expanding and enhancing the functional capacity of CD4 + foxp3 regulatory T cells, thereby inhibiting AS in atherosclerotic-prone mice [37]. We also found that UVB irradiation can reduce the area of atherosclerotic plaques, aortic root plaque area and increase the stability of plaques at the aortic root. We performed UVB irradiation for a short period of time, three times a week for 4 weeks. Studies have shown that the balance of macrophage phenotypes M1 and M2 plays a potential role in plaque stability. M1 leads to fragile plaques, and M2 leads to stable plaques. We showed that UVB could reduce the inflammatory response by distorting macrophage M2 polarization in vivo, and then we determined the vascular protective effect of UVB. In the aortic root plaque in the UVB treatment group, we observed decreased lipid accumulation, decreased macrophage infiltration, and increased collagen and SMCs, suggesting a decrease in the plaque vulnerability index.

In this study, we found that the UVB group mice had lower body weights. This is not the first time we observed this phenomenon. Hart et al. found that UV irradiation significantly reduces weight gain caused by diet [36]. They also found no differences in water, food intake, travel distance, energy expenditure, or respiratory quotient. Perhaps the mechanism of UVB in weight loss needs further research.

On the other hand, by culturing THP-1 cells to induce M0 macrophages in vitro, we found that UVB irradiation induces polarization of M2 macrophages. The results were confirmed by morphological observation, flow cytometry, and western blotting experiments. We further explored the potential mechanism by which UVB promotes the polarization of M2 macrophages in vitro. Akt phosphorylation plays an important role in macrophage polarization. However, the phosphorylation of Akt in M2 polarization is still controversial. The increase in p-Akt promotes M2 polarization of macrophages [31–33]. However, some studies have shown conflicting findings. Decreased p-Akt promotes polarization of M2 macrophages [38]. In our study, we found that an increase in Akt^ser473^ phosphorylation levels promotes the polarization of M2 macrophages, but UVB did not alter phosphorylation of Akt-308 site. An Akt inhibitor (MK-2206 2HCL, Selleck) blocks the effect of UVB on the polarization of M2 macrophages. In Tu et al. study, they found that DNA-PKcs-mTORC2 association is required for UVB-induced Akt Ser-473 phosphorylation and skin keratinocytes survival, maybe this association is also required in THP1 cells. In the following research, we will conduct further exploration and verification [39].

In summary, UVB protected against atherosclerosis by promoting the polarization of M2 macrophages and limiting the inflammatory response in plaques, which was achieved by increasing the phosphorylation of Akt. Our research provides new experimental evidence for the possibility of UVB prevention and treatment of AS. Our results show that UVB light from sunlight has the potential to prevent and treat chronic disease at sites distant from irradiated skin.
